# Capacity of r*Tth* polymerase to detect RNA in the presence of various inhibitors

**DOI:** 10.1371/journal.pone.0190041

**Published:** 2018-01-02

**Authors:** Dongyang Cai, Ole Behrmann, Frank Hufert, Gregory Dame, Gerald Urban

**Affiliations:** 1 Department of Microsystems Engineering (IMTEK), University of Freiburg, Freiburg, Baden-Württemberg, Germany; 2 Institute of Microbiology and Virology, Brandenburg Medical School Theodor Fontane, Neuruppin, Brandenburg, Germany; Meharry Medical College, UNITED STATES

## Abstract

The full potential of the real-time reverse transcription polymerase chain reaction (RT-PCR) as a rapid and accurate diagnostic method is limited by DNA polymerase inhibitors as well as reverse transcriptase inhibitors which are ubiquitous in clinical samples. r*Tth* polymerase has proven to be more resistant to DNA polymerase inhibitors present in clinical samples for DNA detection and also exhibits reverse transcriptase activity in the presence of Mn^2+^ ions. However, the capacity of r*Tth* polymerase, which acts as DNA polymerase and reverse transcriptase, to detect RNA in the presence of various inhibitors has not been investigated in detail. Herein, the inhibitors originating from various clinical samples such as blood, urine, feces, bodily fluids, tissues and reagents used during nucleic acid extraction were employed to evaluate the capacity of r*Tth* polymerase to detect RNA. The results show that the inhibitors have different inhibitory effects on the real-time RT-PCR reactions by r*Tth* polymerase, and the inhibitory effects are concentration dependent. Additionally, the capacity of r*Tth* polymerase to detect RNA in the presence of various inhibitors is better or at least comparable with its capacity to detect DNA in the presence of various inhibitors. As a consequence, RNA may be directly detected in the presence of co-purified inhibitors or even directly from crude clinical samples by r*Tth* polymerase.

## Introduction

The real-time reverse transcription polymerase chain reaction (RT-PCR) is a useful and important technology in the clinical diagnostic laboratory, which has been widely used for the measurement of gene expression, detection of cancer, diagnosis of infectious agents or genetic diseases [1–4]. Theoretically, real-time RT-PCR differs from real-time PCR only by the addition of an initial step which converts RNA into complementary DNA by a reverse transcriptase. The co-purified inhibitors from clinical samples are highly inhibitory to real-time PCR and the DNA polymerase is the most likely target site of these inhibitors [5]. The reverse transcriptase used in the RT step is another target site of the inhibitors and its resistance may differ from the DNA polymerase, resulting in more assay variation [6–10].

A number of clinically relevant substances have been reported to be DNA polymerase inhibitors, namely, lactoferrin, hemoglobin, immunoglobulin G (IgG) and added anticoagulants in blood, myoglobin in muscle tissues, urea in urine, bile salts in feces, some metal ions in bodily fluids and tissues, and reagents used during nucleic acid extraction [11–16]. Additionally, some reverse transcriptases such as Moloney-murine leukemia virus reverse transcriptase and Rous-associated virus 2 reverse transcriptase have been reported to be inhibited by some substances co-purified from clinical samples [6–10]. For example, when the HIV-1 RNA was detected by RT-PCR in the presence of co-purified heparin, only 26% samples showed positive results [8]. Heparin is extremely hard to remove because it can co-purify with the RNA throughout numerous isolation procedures, even those using column purification [17]. When applying the real-time RT-PCR technique to RNA extracted from clinical samples, it must be considered that the co-purified inhibitors may negatively affect the sensitivity of the assay or even cause false-negative results. Foreign RNAs are commonly used in real-time RT-PCR as internal controls to indicate the presence of inhibitors and normalize the variation caused by these substances [18, 19]. The detection of target and control sequences are inhibited to the same extent is the prerequisite of using such controls, however, which is not always the case [20]. When the presence of co-purified inhibitors is suspected, investigators tend to dilute or re-purify the RNA sample when the abundance of the target is not limiting. An easier and more reliable way is to use reverse transcriptase and DNA polymerase that are more resistant to inhibitors.

r*Tth* polymerase, derived from the eubacterium *Thermus thermophilus* HB8, has proven to be more resistant to DNA polymerase inhibitors present in clinical samples for DNA detection and also exhibits reverse transcriptase activity in the presence of Mn^2+^ ions [21, 22]. The capacity of r*Tth* polymerase to detect DNA in the presence of different concentrations of Na^+^ ions, Ca^2+^ ions, Fe^3+^ ions, EDTA, heparin, bile salts, IgG, lactoferrin, hemoglobin, and myoglobin has been reported [11–13, 21]. There is also evidence that RNA detection by r*Tth* polymerase is more resistant to the inhibitors present in nasopharyngeal swab compared to *Taq* polymerase [23]. However, the full potential of r*Tth* polymerase to detect RNA in the presence of other clinically relevant inhibitors has not been reported.

In this study, we investigated the capacity of r*Tth* polymerase which acted as both reverse transcriptase and DNA polymerase to detect RNA in the presence of different concentrations of blood, tissue, feces and bodily fluids originating inhibitors as well as the reagents used during nucleic acid extraction. The results show that a) the inhibitors have different inhibitory effects on the real-time RT-PCR reactions by r*Tth* polymerase, and the inhibitory effects are concentration dependent; b) the capacity of r*Tth* polymerase to detect RNA in the presence of various inhibitors is better or at least comparable with its capacity to detect DNA in the presence of various inhibitors; c) certain RNA samples with co-purified inhibitors can be detected directly by r*Tth* polymerase without the need for dilution or re-purification; d) RNA may be detected directly from certain clinical samples by r*Tth* polymerase in the presence of RNase inhibitors.

## Materials and methods

### Bacterial culture and RNA extraction

*Enterococcus faecalis* Symbioflor 1 (*E*. *faecalis* Symbioflor 1) was cultured overnight in lysogeny broth Luria (LB-Luria). 1 ml bacterial culture was centrifuged at 8000×g for 5 min to pellet the bacteria. The supernatant was discarded and the pellet was washed one time with distilled water. *E*. *faecalis* Symbioflor 1 total RNA was extracted using the RNeasy Mini Kit (Qiagen, Hilden, Germany). All extraction procedures were performed according to the manufacturer’s instructions. DNase I (Ambion, Kaufungen, Germany) treatment was used to remove trace amounts of genomic DNA from the total RNA. The final concentration of the total RNA (19.6 ng/uL) was determined spectrophotometrically using a NanoPhotometer (Implen, Munchen, Germany).

### PCR inhibitory samples

Different concentrations of NaCl (Sigma, Steinheim, Germany), CaCl_2_·2H_2_O (Sigma, Neu-Ulm, Germany), FeCl_3_·6H_2_O (Merck, Darmstadt, Germany), disodium EDTA·2H_2_O (Sigma, Steinheim, Germany), trisodium citrate·2H_2_O (Sigma, Vienna, Austria), sodium heparin (Novo, Vienna, Austria), sodium dodecyl sulfate (SDS, Merck, Darmstadt, Germany), proteinase K (Ambion, Kaufungen, Germany), urea (Sigma, Steinheim, Germany), bile salts (Sigma, Steinheim, Germany), hemoglobin and lactoferrin from human blood (Sigma, Steinheim, Germany), bovine IgG (Sigma, Steinheim, Germany), and myoglobin from human heart (Sigma, Steinheim, Germany) were prepared in DEPC treated water to investigate their effects on r*Tth* polymerase to detect RNA. The final concentrations in the real-time RT-PCR mixtures were 10, 20, 40, 60, 80, 100 mM NaCl; 6, 8, 10, 12, 14, 16 mM CaCl_2_; 0.01, 0.1, 0.5, 1, 5, 10 mM FeCl_3_; 1, 2, 4, 6, 8, 10 mM EDTA; 1, 5, 10, 20, 40, 60 mM citrate; 1, 5, 10, 20, 40, 60 IU heparin; 10, 20, 50, 100, 150, 200 μg/ml SDS; 5, 10, 20, 40, 60, 80 μg/ml proteinase K; 50, 100, 150, 200, 300, 400 mM urea; 10, 50, 100, 200, 500, 1000 μg/ml bile salts; 5, 10, 20, 50, 100, 200 μg/ml lactoferrin; 0.1, 0.2, 0.5, 1, 2, 5 mg/mL hemoglobin; 10, 20, 50, 100, 200, 300 μg/ml IgG; 30, 60, 90, 120, 300, 600 μg/ml myoglobin.

### RT-PCR assay

The volume of the real-time RT-PCR mixture was 20 μL. The assay was performed with forward primer 5’-TGAATTGCGTTTCGTAGGTTAC-3’, reverse primer 5’-CCAAACATATTGCCACTTAAATCTC-3’ and Taqman probe 5’-TCGGGTCAGGGTCCTAATCGAAGTGG-3’ which were designed targeting the CNRZ16 gene of *E*. *faecalis* Symbioflor 1 using the Primer3plus online service. All of the real-time RT-PCR mixtures contained 1×RT-PCR buffer (50 mM bicine/KOH, 115 mM K-acetate, 8% v/v glycerol), 0.5 μM of each primer, 0.2 mM of each dNTP, 4 mM MnCl_2,_ 0.3 μM Taqman probe and 0.25 U/μL r*Tth* polymerase (Bioron, Ludwigshafen, Germany). Varying amounts of inhibitory substances were added to the real-time RT-PCR mixtures to obtain the final concentrations. 1 μL *E*. *faecalis* Symbioflor 1 total RNA (19.6 ng/μL) was added to the real-time RT-PCR mixtures as the last ingredient. The RT step was performed at 61 ºC for 30 min, the following PCR step was performed at 94 ºC for 2 min, followed by 50 thermal cycles at 94 ºC for 15 s, 60 ºC for 45 s, and a final extension at 72 ºC for 2 min. The results were then presented by amplification curves and end-point fluorescence intensity ratios: (fluorescence intensity at cycle 40 in the presence of inhibitors **/** fluorescence intensity at cycle 40 in the absence of inhibitors) **×** 100. After 40 thermal cycles, the amplification curve of positive control reached the plateau phase, whereas the fluorescence intensity of the PCR reactions in the presence of certain inhibitors continued to slightly increase even at cycle 50. The additional amplification after 40 cycles in the presence of inhibitors can mask the decreased capacity of r*Tth* polymerase for RNA detection. Therefore, the fluorescence intensity at cycle 40 was chosen to plot the fluorescence intensity ratios. All these experiments were repeated for three times.

## Results

### Inhibitory effects of metal ions on the RNA detection by r*Tth* polymerase

**Fig 1:**
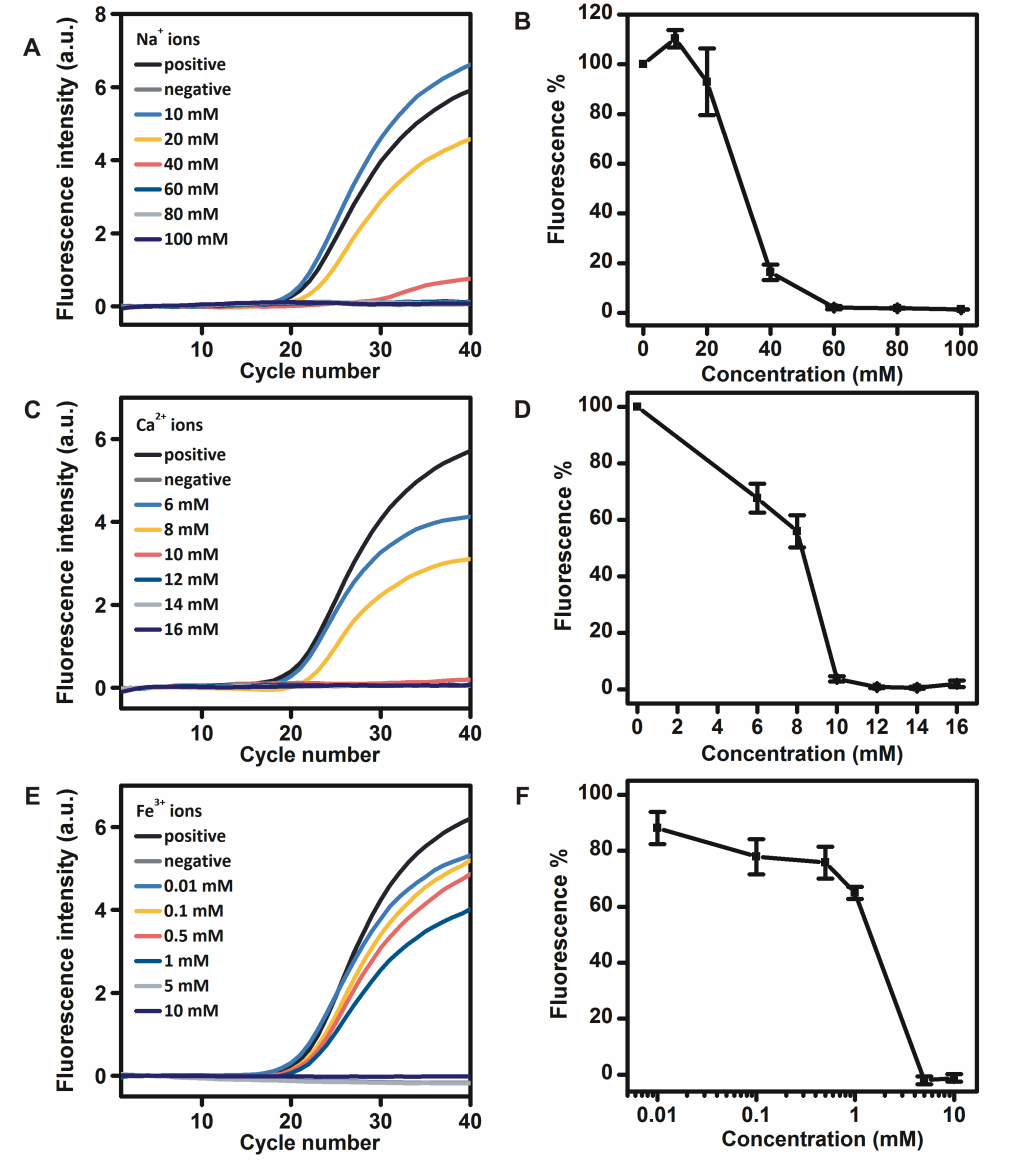
Inhibitory effects of different concentrations of Na^+^ ions A, B), Ca^2+^ ions C, D), and Fe^3+^ ions E, F) on the RNA detection by r*Tth* polymerase.

Metal ions are present in a variety of clinical samples and their inhibitory effects on r*Tth* polymerase to detect DNA have been well described [11, 21]. Different concentrations of Na^+^, Ca^2+^ and Fe^3+^ ions were added to the real-time RT-PCR mixtures to investigate their effects on the RNA detection by r*Tth* polymerase. The addition of Na^+^ ions at a concentration of 10 mM in the real-time RT-PCR mixture increased the fluorescence intensity to approximately 110.3%, whereas the addition of Na^+^ ions at concentrations of 20 and 40 mM in the real-time RT-PCR mixtures reduced the fluorescence intensity to approximately 93.0% and 16.4%, respectively. The addition of Na^+^ ions at concentrations of 60, 80 and 100 mM in the real-time RT-PCR mixtures completely inhibited the RNA detection by r*Tth* polymerase (Fig 1A and 1B). The addition of Ca^2+^ ions at concentrations of 6 and 8 mM in the real-time RT-PCR mixtures reduced the fluorescence intensity to approximately 67.7% and 55.9%, respectively. The addition of Ca^2+^ ions at concentrations of 10, 12, 14 and 16 mM in the real-time RT-PCR mixtures completely inhibited the RNA detection by r*Tth* polymerase (Fig 1C and 1D). The addition of Fe^3+^ ions at concentrations of 0.01, 0.1, 0.5 and 1 mM in the real-time RT-PCR mixtures reduced the fluorescence intensity to approximately 88.1%, 77.8%, 75.8% and 65.0%, respectively. The addition of Fe^3+^ ions at concentrations of 5 and 10 mM in the real-time RT-PCR mixtures completely inhibited the RNA detection by r*Tth* polymerase (Fig 1E and 1F). It was found that the higher valence of ions were added, the more significant inhibition was observed. The inhibitory effects of trivalent ions were more significant than divalent ions and those of divalent ions were more significant than monovalent ions.

### Inhibitory effects of anticoagulants on the RNA detection by r*Tth* polymerase

**Fig 2:**
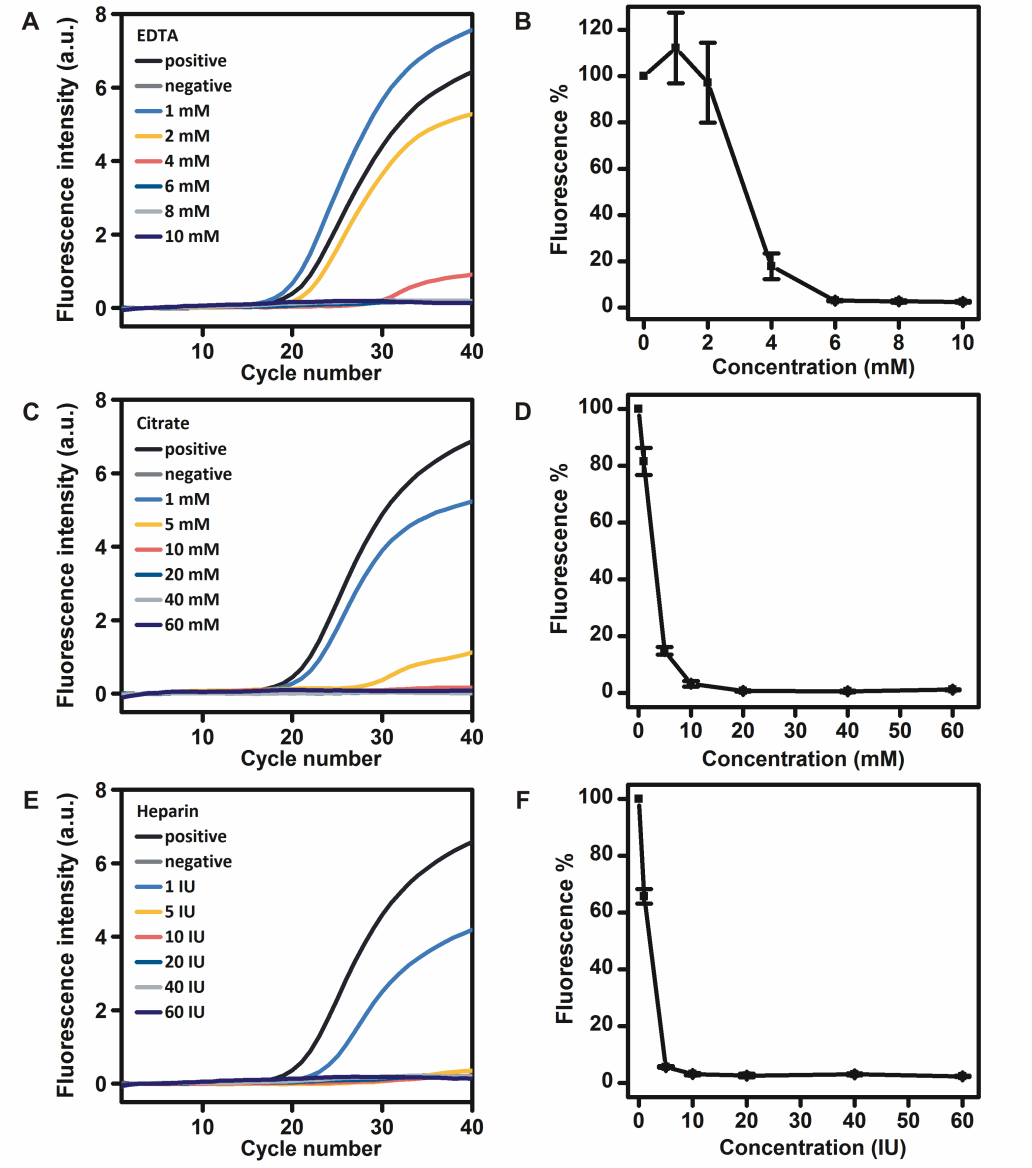
Inhibitory effects of different concentrations of EDTA A, B), citrate C, D), and heparin E, F) on the RNA detection by r*Tth* polymerase.

In the medical laboratory, EDTA, citrate and heparin are commonly used for the purpose of blood anticoagulation. Different concentrations of EDTA, citrate and heparin were added to the RT-PCR mixtures to investigate their effects on the RNA detection by r*Tth* polymerase. The addition of EDTA at a concentration of 1 mM in the real-time RT-PCR mixture increased the fluorescence intensity to approximately 112.0%, whereas the addition of EDTA at concentrations of 2 and 4 mM in the real-time RT-PCR mixtures reduced the fluorescence intensity to approximately 97.2% and 17.8%, respectively. The addition of EDTA at concentrations of 6, 8 and 10 mM in the real-time RT-PCR mixtures completely inhibited the RNA detection by r*Tth* polymerase (Fig 2A and 2B). The addition of citrate at concentrations of 1 and 5 mM in the real-time RT-PCR mixtures reduced the fluorescence intensity to approximately 81.5% and 14.8%, respectively. The addition of citrate at concentrations of 10, 20, 40 and 60 mM in the real-time RT-PCR mixtures completely inhibited the RNA detection by r*Tth* polymerase (Fig 2C and 2D). The addition of heparin at a concentration of 1 IU in the real-time RT-PCR mixtures reduced the fluorescence intensity to approximately 65.7%. The addition of heparin at concentrations of 5, 10, 20, 40, 60 IU in the real-time RT-PCR mixtures completely inhibited the RNA detection by r*Tth* polymerase (Fig 2E and 2F).

### Inhibitory effects of bile salts, urea, SDS, and proteinase K on the RNA detection by r*Tth* polymerase

**Fig 3:**
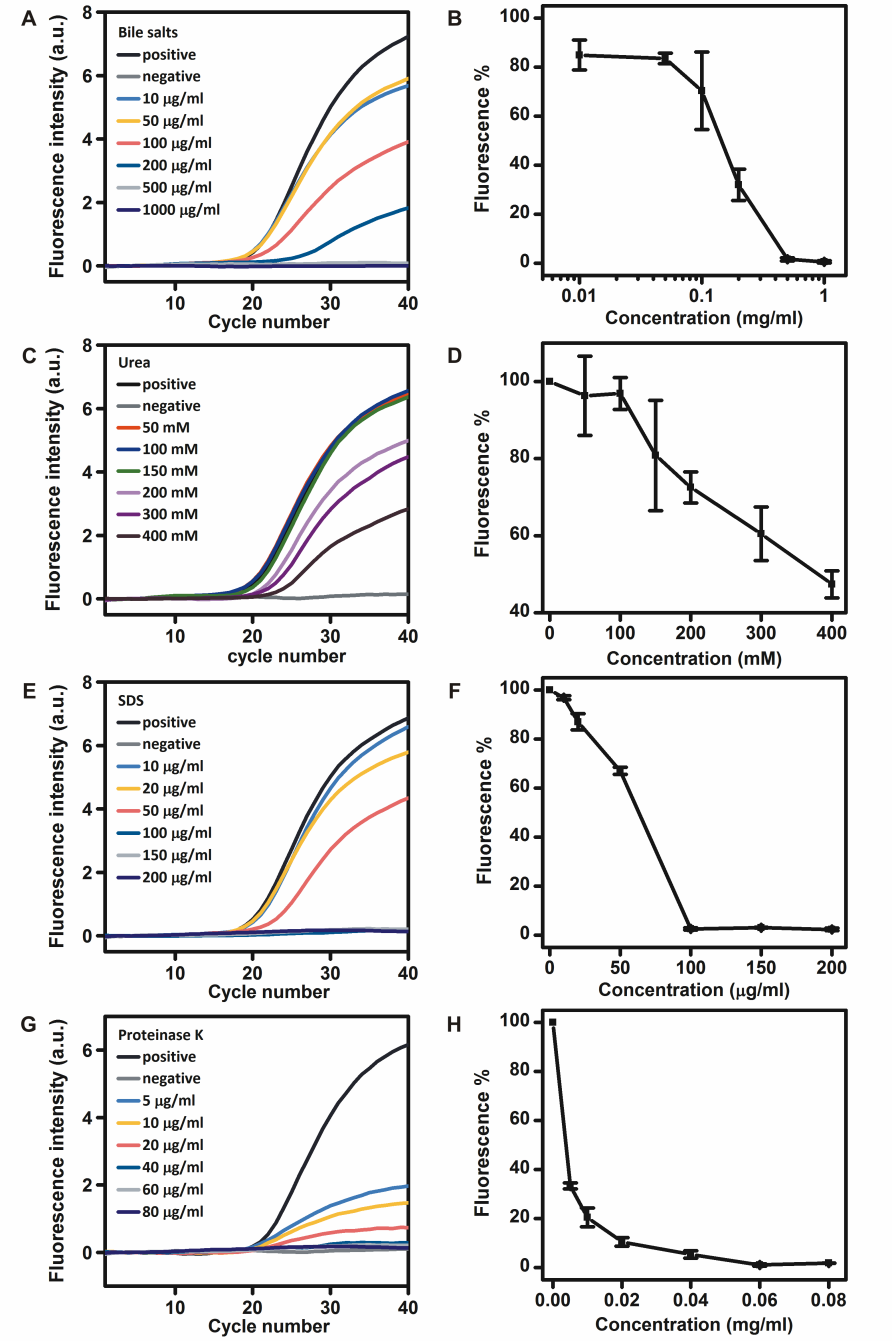
Inhibitory effects of different concentrations of bile salts A, B), urea C, D), SDS E, F), and proteinase K G, H) on the RNA detection by r*Tth* polymerase.

Bile salts and urea are well-known DNA polymerase inhibitors in feces and urine, respectively [14, 15]. Different concentrations of bile salts and urea were added to the real-time RT-PCR mixtures to investigate their effects on the RNA detection by r*Tth* polymerase. The addition of bile salts at concentrations of 10, 50, 100 and 200 μg/ml in the real-time RT-PCR mixtures reduced the fluorescence intensity to approximately 84.9%, 83.5%, 70.4%, and 32.0%, respectively. The addition of bile salts at concentrations of 500 and 1000 μg/ml in the real-time RT-PCR mixtures completely inhibited the RNA detection by r*Tth* polymerase (Fig 3A and 3B). The addition of urea at concentrations of 50, 100, 150, 200, 300 and 400 mM in the real-time RT-PCR mixtures reduced the fluorescence intensity to approximately 96.3%, 96.9%, 80.8%, 72.6%, 60.5% and 47.4%, respectively (Fig 3C and 3D).

SDS and proteinase K are necessary for efficient cell lysis to prepare pure nucleic acids, but the residual reagents may also cause inhibitory effects [16]. Different concentrations of SDS and proteinase K were added to the real-time RT-PCR mixtures to investigate their effects on the RNA detection by r*Tth* polymerase. The addition of SDS at concentrations of 10, 20 and 50 μg/ml in the real-time RT-PCR mixtures reduced the fluorescence intensity to approximately 96.8%, 87.0% and 67.0%, respectively. The addition of SDS at concentrations of 100, 150 and 200 μg/ml in the real-time RT-PCR mixtures completely inhibited the RNA detection by r*Tth* polymerase (Fig 3E and 3F). The addition of proteinase K at concentrations of 5, 10, and 20 μg/ml in the real-time RT-PCR mixtures reduced the fluorescence intensity to approximately 33.3%, 20.5% and 10.5%, respectively. The addition of proteinase K at concentrations of 40, 60 and 80 μg/ml in the real-time RT-PCR mixtures completely inhibited the RNA detection by r*Tth* polymerase (Fig 3G and 3H).

### Inhibitory effects of proteins in clinical samples on the RNA detection by r*Tth* polymerase

**Fig 4:**
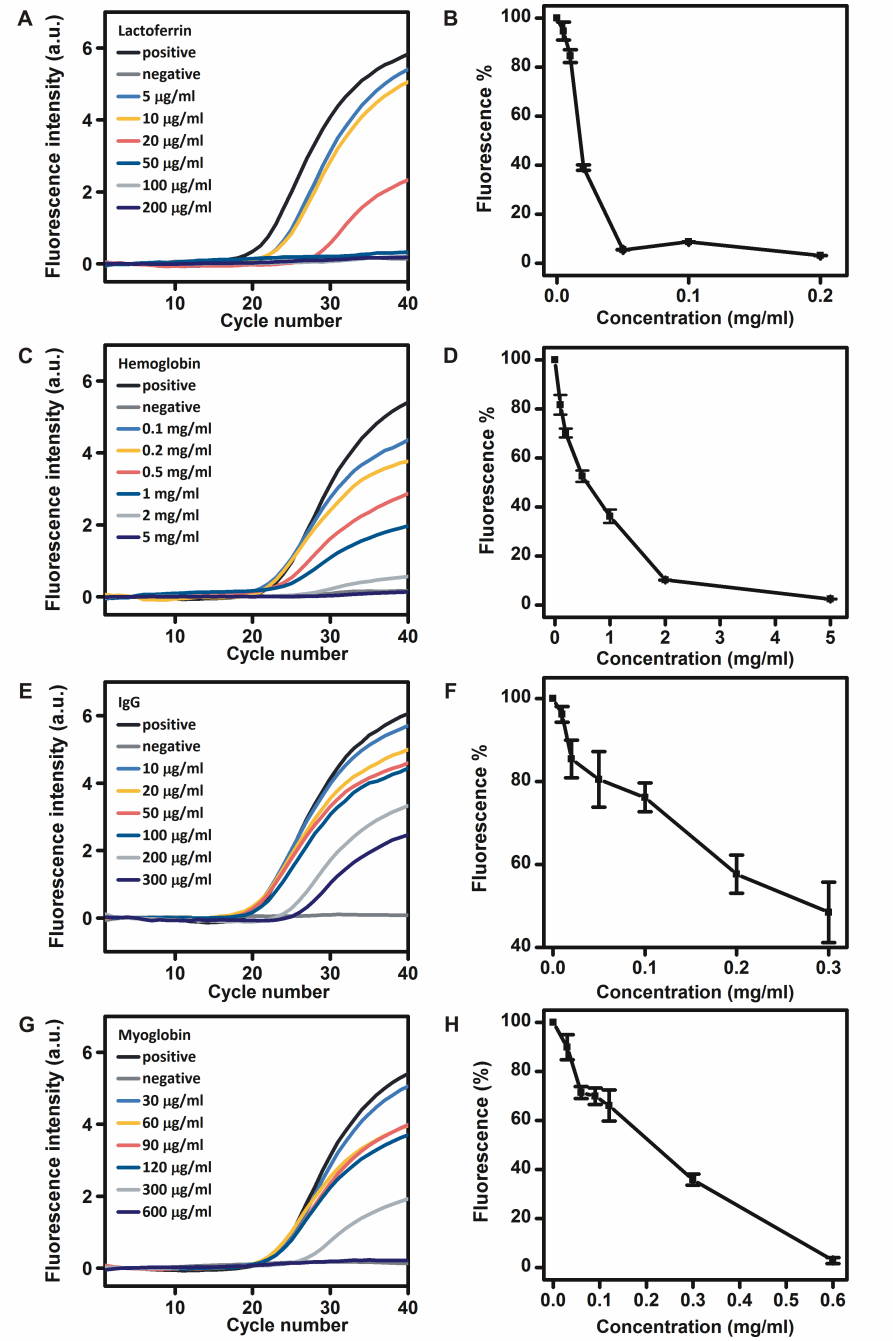
Inhibitory effects of different concentrations of lactoferrin A, B), hemoglobin C, D), IgG E, F), and myoglobin G, H) on the RNA detection by r*Tth* polymerase.

Lactoferrin in leukocytes, hemoglobin in erythrocytes, IgG in plasma and myoglobin in muscle tissues have been reported to be PCR inhibitors [11–13]. Different concentrations of lactoferrin, hemoglobin, IgG and myoglobin were added to the RT-PCR mixtures to investigate their effects on the RNA detection by r*Tth* polymerase. The addition of lactoferrin at concentrations of 5, 10 and 20 μg/ml in the real-time RT-PCR mixtures reduced the fluorescence intensity to approximately 94.7%, 84.5% and 39.0%, respectively. The addition of lactoferrin at concentrations of 50, 100 and 200 μg/ml in the real-time RT-PCR mixtures completely inhibited the RNA detection by r*Tth* polymerase (Fig 4A and 4B). The addition of hemoglobin at concentrations of 0.1, 0.2, 0.5, 1 and 2 mg/ml in the real-time RT-PCR mixtures reduced the fluorescence intensity to approximately 81.7%, 70.2%, 52.5%, 36.2% and 10.2%, respectively. The addition of hemoglobin at a concentration of 5 mM completely inhibited the RNA detection by r*Tth* polymerase (Fig 4C and 4D). The addition of IgG at concentrations of 10, 20, 50, 100, 200, and 300 μg/ml in the real-time RT-PCR mixtures reduced the fluorescence intensity to approximately 96.2%, 85.4%, 80.5%, 76.2%, 57.7%, and 48.4%, respectively (Fig 4E and 4F). The addition of myoglobin at concentrations of 30, 60, 90, 120, and 300 μg/ml in the real-time RT-PCR mixtures reduced the fluorescence intensity to approximately 89.9%, 71.4%, 69.9%, 66.1% and 35.8%, respectively. The addition of myoglobin at a concentration of 600 μg/ml in the real-time RT-PCR mixture completely inhibited the RNA detection by r*Tth* polymerase (Fig 4G and 4H).

## Discussion

Metal ions in bodily fluids and tissues, anticoagulants in blood, reagents used during nucleic acids extraction, organic substances in urine and feces and proteins in blood and muscle tissues were used to evaluate the capacity of r*Tth* polymerase to detect RNA. The study demonstrates that the inhibitors have different inhibitory effects on the real-time RT-PCR reactions by r*Tth* polymerase, and these effects are concentration dependent. Additionally, the capacity of r*Tth* polymerase to detect RNA in the presence of various inhibitors is better or at least comparable with its capacity to detect DNA in the presence of various inhibitors. As a consequence, RNA may be directly detected in the presence of co-purified inhibitors or even directly from crude clinical samples by r*Tth* polymerase.

K^+^ ions in the PCR buffer are used to ensure suitable ion content. The addition of Na^+^ ions may disturb the ion content and therefore affect both polymerase activity and primer annealing conditions [24]. It has been reported that Na^+^ ions at a concentration of 150 mM were completely inhibitory to DNA detection by r*Tth* polymerase [21]. Standard ion content needed by r*Tth* polymerase for RNA detection may explain its higher resistance to Na^+^ ions for DNA detection. It has been reported that the addition of Ca^2+^ ions at a concentration of 2 mM in the PCR mixture was completely inhibitory to the DNA detection by r*Tth* polymerase and the addition of Fe^3+^ ions at a concentration higher than 25 μM inhibited the DNA detection by r*Tth* to less than 10% [11, 21]. It has also been reported that the addition of 0.25 mM EDTA and 0.01 IU heparin inhibited the DNA detection by r*Tth* to about 46% and 51%, respectively [11]. Ca^2+^ ions, Fe^3+^ ions, EDTA, and citrate inhibit PCR by competing with or chelating Mg^2+^ ions which are necessary for r*Tth* polymerase activity. The inhibitory effect of heparin has been reported on the basis of an interaction with DNA and DNA polymerase. Although both DNA and heparin are highly negatively charged, and would not be expected to interact, binding between these two molecules could be mediated by divalent cations such as Mg^2+^ ions [25]. Thus, a higher concentration of Mn^2+^ ions (4 mM) was used as a cofactor of r*Tth* polymerase for RNA detection instead of Mg^2+^ ions, resulting in higher resistance to Ca^2+^ ions, Fe^3+^ ions, EDTA, citrate, and heparin. Additionally, Mn^2+^ ions have been reported to bind with DNA polymerase more tightly compared with Mg^2+^ ions, which is another possible explanation for the high resistance of r*Tth* polymerase to detect RNA [26]. RNA samples with these co-purified inhibitors may thus be detected directly by r*Tth* polymerase.

SDS, proteinase K and urea act as PCR inhibitors by degradation of DNA polymerases [5]. r*Tth* polymerase was able to detect RNA in the presence of 100 mM urea without reduced detection capacity. The special protein structure of r*Tth* polymerase is one possible explanation of its higher resistance to urea degradation. The urea excretion for an adult human is dependent on diet and age which is in the range between 340 and 580 mmol per day [5]. Therefore urine may be directly analyzed without any sample preparation procedures by r*Tth* polymerase in the presence of RNase inhibitors. Bile salts are polar derivatives of cholesterol and contain both polar and non-polar regions [15]. The mechanism of bile salts as PCR inhibitors is still unknown. It has been reported that the addition of 0.25 to 0.1 mg/ml bile salts reduced the DNA detection by r*Tth* polymerase to about 76%, which is very similar to our results for RNA detection [11].

Lactoferrin, hemoglobin and myoglobin have been reported to be the major PCR inhibitors in leukocytes, erythrocytes, and muscle tissues. The mechanism of PCR inhibition by these substances may be related to their abilities to release Fe^3+^ ions. The derivatives of hemoglobin (bilirubin, bile salts, and heme) and myoglobin (heme) were also found to be PCR inhibitors [11, 13]. Lactoferrin has been reported to interact with nucleic acids, which is another possible mechanism of PCR inhibition [12]. IgG in blood plasma forms a complex with ssDNA, therefore affects primer annealing and the concentration of free primers. It has been reported that r*Tth* polymerase was able to detect DNA in the presence of 0.164 mg/mL IgG, which is very similar to our results for RNA detection [12]. It has also been reported that r*Tth* polymerase enabled to detect DNA in the presence of 1 μg/mL lactoferrin and 4 mg/mL hemoglobin [11]. The higher resistance of lactoferrin in our study is probably because higher concentrations of Mn^2+^ ions and different experimental conditions were used. The lower resistance of hemoglobin in our study may be due to its fluorescence quenching capacity, which was further demonstrated by a conventional PCR reaction. In that reaction, r*Tth* polymerase enabled RNA detection in the presence of at least 40 mg/mL hemoglobin (S1 Fig).

Significantly, the error rate of r*Tth* polymerase is increased in the presence of Mn^2+^ ions [27]. When high fidelity is required in the RT-PCR products, a two step RT-PCR is recommended. The RT step is performed to reverse transcribe RNA by r*Tth* polymerase in the presence of Mn^2+^ ions and the PCR step is performed in the same tube simply by chelation of Mn^2+^ ions and the addition of Mg^2+^ ions. Additionally, the inhibitory effects of inhibitors on reverse transcriptase or DNA polymerase can be determined using a two step RT-PCR.

## Supporting information

S1 Fig RNA detection in the presence of different concentrations of hemoglobin by rTth polymerase. (TIF)
