# Supplementary figures and images for: Capacity of rTth polymerase to detect RNA in the presence of various inhibitors
Source: PLoS One. 2018 Jan 2;13(1):e0190041. doi: 10.1371/journal.pone.0190041 (PMC5749758; doi:10.1371/journal.pone.0190041)

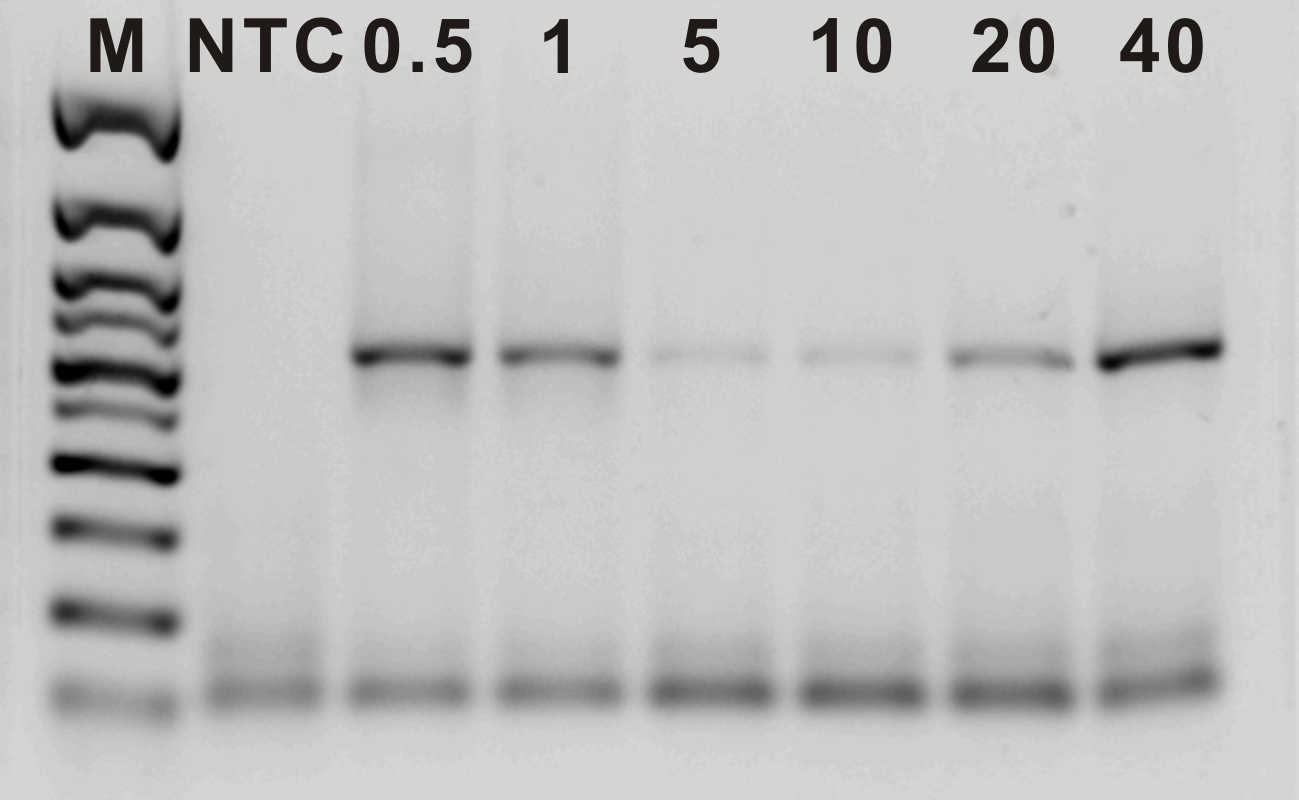

Supplement: S1 Fig — (TIF) [file pone.0190041.s001.tif]
